# The epidemiology of oral cancer during the COVID-19 pandemic in Northern Italy: Incidence, survival, prevalence

**DOI:** 10.3389/froh.2022.982584

**Published:** 2022-09-16

**Authors:** Lucia Mangone, Pamela Mancuso, Isabella Bisceglia, Giacomo Setti, Giuliano Malaguti, Paolo Giorgi Rossi

**Affiliations:** ^1^ Epidemiology Unit, Azienda Unità Sanitaria Locale – IRCCS di Reggio Emilia, Reggio Emilia, Italy; ^2^ Centro Universitario Odontoiatria, University of Parma, Parma, Italy; ^3^ Department of Specialist Surgeries Head-Neck, Modena and Reggio Emilia University, Modena, Italy

**Keywords:** oral cancer, incidence, survival, prevalence, COVID-19

## Abstract

Despite novel treatment approaches, oral cancer survival has not improved significantly and the disease often presents a disabling path for patients. The aim of this work was to describe the epidemiological data of oral cancers in a province of northern Italy. Incident cases in the period 1996–2020 and EU population standardized rates were reported for Oral Cavity cancer (OC) and OroPharyngeal cancer (OP). Annual percent changes (APC) were estimated with joint point analysis. The 5-year survival was calculated in three different periods: 1996–2000, 2001–2010 and 2011–2015. From 1996 to 2020, 771 cases of oral cancers (442 OC and 329 OP) were recorded with the age-standardized incidence rate 7.28 (10.74 in males and 3.97 in females): 3.82 for OC and 3.47 for OP. In males there is a significant increase in the incidence of OP up to 2017 (APC 11; 95% CI, 4.9–17.5), which then decreases; in females the rates are constant. In 2020 (the era of Covid-19), we did not see a decline in incidence compared to 2019. The 5-year survival (for cases diagnosed in 2011–2015) was 55.6%, 56.5% and 56% for OC, OP and OC + OP, respectively; it was somewhat higher in females and was undergoing some changes over the course of years. The number of prevalent cases as of 1 January 2021 is 314 (175 OC, 139 OP). The study showed a decline in cancers in men, particularly for OP; survival shows improvement in the long-term examined; Covid-19 had no negative impact on 2020 diagnoses.

## Introduction

Tumors of the oral cavity are infrequent neoplasms but they represent an underestimated public health problem (1). Incidence rate considering all subsites together (oral cavity including lips, pharyngeal region, ICD_O III edition C00–C14) (2) is about 500,000 yearly cases globally. Oral cavity and oropharynx are contiguous organs with distinct embryological origin and proven differences in cancer development (3). Confusion in groupings due to variations in subsites attribution is affecting the literature. Therefore, a quantitative analysis of disease relies on clear and uniform definitions which include topography (site), morphology (histopathology), and tumor behavior (invasive—malignant, benign, in-situ). To date, there is a large consensus that oral cancer should be recognized as being two distinct diseases: Oral Cavity cancer (OC) and OroPharyngeal cancer (OP) (4). This clarification is necessary to better understand the attributable fraction to HPV (Human Papilloma Virus) infection (5), alongside the well-known risk factors such as smoking and alcohol (6).

The association with risk factors has been known for some time: in fact, tumors of the oral cavity are largely linked to the consumption of alcohol and tobacco smoke (7–9) while tumors of the oropharynx are largely linked to HPV infection (10–12).

In addition to exposure to risk factors, the sharing of morphology also unites these tumors: squamous cell carcinoma (SCC) accounts for 90%–95% of OCs (13). It may arise ex novo or from a potentially malignant disorder (PMD, lesions with increased risk of cancer development that can exhibit different grade of dysplasia) and usually spreads locally invading surrounding structures.(14) The lower lip, oral tongue, floor of the mouth and retro molar trigone are the malignancy outbreak site in more than 75% of SCC cases. Disease biological behavior directly affects progression and prognosis worsening. Extension and depth of invasion define the T stage according to TNM classification, nodes metastases represent a pitfall for prognosis (15). The survival rate drops dramatically from about 90% to 25%–40% at 5-years when loco-regional nodes are involved (3). Known risk factors are smoking, alcohol drinking and HPV (16–20). Incidence peaks after the age of 70 and it is much higher in males (13).

OP commonly arise in the tonsils and the tongue base, with a poorly differentiated form of SCC, locally advanced at the time of clinical presentation (14). Known risk factors are the same as OC, but HPV infection is responsible for a larger proportion of cancers in OC than in OP. Worldwide, the estimated HPV-attributable portion of oropharyngeal cancer is less than 30% but numbers are expected to increase (16). Patients with HPV-positive OP are more likely to present with small primary tumors and extensive nodal disease. Even with the early nodes involvement, 5-year survival rate is higher in such patients compared to HPV-negative cases (17). HPV-positive cancers usually occur in younger male patients, and the role of smoking and alcohol as risk factors is smaller.

Even if there are still some critical issues regarding risk factor awareness among the target population, it is important to increase data sharing and knowledge, to help improve the level of awareness and assessment of risk factors for oral cancer (21).

In Italy there are no epidemiological data on the incidence of oral cancers and in particular there is a lack of recent data. The estimates to 2018 are projections of the incident years in 2003–2014: incidence and mortality decrease in males and appear to slightly increase in females (22).

The aim of this work is to describe the incidence data, survival and prevalence of oral cancer in a province of northern Italy and, with the availability of data for 2020, also evaluate the impact of Covid-19 on new cancer diagnoses.

## Materials and methods

### Data source

This is a population-based cohort study using data from the Reggio Emilia Cancer Registry (RE-CR) approved by the provincial Ethics Committee of Reggio Emilia (Protocol n. 2014/0019740 of 04/08/2014). Reggio Emilia is a province in northern Italy with about 532,000 inhabitants.

The RE-CR registers all new cancer diagnoses occurring in people residing in the Reggio Emilia Province. The main information sources are the anatomic pathology reports, the hospital discharge records, and mortality data from the RE-CR. The RE-CR, which covers the entire resident population in the Province of Reggio Emilia, has been active since 2000 and registered all incident cases from 1996 to 2020, with an active follow up for deaths and residence of all prevalent cases updated up to 01/01/2021. It collects information on site, morphology, type of diagnosis, survival and prevalence (23).

### Definitions of oral cancer

According to the International Classification of Diseases in Oncology (ICD-O III edition) we have classified tumors using the following criteria*. Oral cavity cancer* includes: the inner lip (C00.3–C00.9), other and unspecified parts of the tongue (C02) (excluding lingual tonsil [C2.4]), gum (C03), floor of the mouth (C04), palate (C05), and other and unspecified parts of the mouth (C06) (2).

To allow easy comparison with other recent data, we grouped oral cancers according to a definition proposed by Conway (4). *Oropharyngeal cancer* includes: the base of the tongue (C01), lingual tonsil (C2.4), tonsil (C09), oropharynx (C10), and pharynx unspecified including Waldeyer's ring/overlapping sites of oral cavity and pharynx (C14).

In addition to the classification by site, the morphologies of the tumors have been defined, as reported in the ICD-O III edition classification, in this case the tumors include all the indicated morphologies, including for example the NHL of the Waldayer's ring.

### Data analyses

Only infiltrating malignant tumors from subjects residing in the province of Reggio Emilia at the time of diagnosis were included, in accordance with national (24) and international rules (25).

The registration rules do not provide for the inclusion of *in situ* tumors, benign and with uncertain behavior (except for cancers of the bladder and central nervous system): the standardization of the rules allows CRs to easily compare cases incident in various parts of the world. Descriptive analyses of patient characteristics were performed for OP and OC cancers. Incidence (age-adjusted on European Standard Population 2013) rate data for 2019 and 2020, and incidence trends for the period 1996–2020 are reported; this is done separately for males and females and for OP and OC cancers.. Trends over time were analyzed by calculating the annual percent change (APC) in age-standardized rates using Joinpoint Regression (26).

Incident, cases from 1996 to 2015 were used to calculate survival and were divided into three periods (1996–2000, 2001–2010, 2011–2015). The Kaplan–Meier method was used to trace survival over a 5-year period for OP and OC cancers, sex and period with the aim of assessing whether anything had changed during the three periods examined. The analyzes are shown in the graphs, and the values are also expressed in the table.

Prevalence was estimated on January 1, 2021, for years by diagnosis (<2, 2–9, ≥9), by subtype and sex. StataSE v. 16.1 (StataCorp LP, College Station, TX) was used for all analyses.

## Results

From 1996 to 2020, 771 cases of oral cancers, 442 OC and 329 OP were recorded. The average age is around 65.5 years, it affects males more, with a M: F ratio of 2:1. In the study period, divided into five years, there was an increase in cases: it went from 107 in 1996–2000 to 174 in 2016–2020. The sites for which a higher number of cases are recorded are: for OC, the dorsal surface of the tongue (124 cases), mouth (86 cases) and overlapping lesions of the tongue (53 cases); for OP the majority of cases are recorded for the tonsils (184 cases) (Table 1).

**Table 1:** Distribution of 771 patients with oral cancer, by age, sex, years of diagnosis, site and morphology.

	Oral cavity cancer		Oropharyngeal cancer		Total
	*n*	%	*n*	%	*n*
Overall	442		329		771
Age (mean. sd)	66.9 (14.4)		63.5 (13.1)		65.5 (13.9)
Sex					
Male	279	63.1	233	70.8	512
Female	163	36.9	96	29.2	259
Year					
1996–2000	66	14.9	41	12.5	107
2001–2005	89	20.1	60	18.2	149
2006–2010	87	19.7	96	29.2	183
2011–2015	97	21.9	61	18.5	158
2016–2020	103	23.3	71	21.6	174
Site					
Lip (C00.3–C00.9)	28	6.3			
Dorsal surface of tongue. NOS (C02.0–C02.3)	124	28.1			
Overlapping lesion of tongue. or tongue NOS (C02.8–C02.9)	53	12.0			
Gum (C03)	41	9.3			
Floor of mouth (C04)	45	10.2			
Soft palate (C05.1)	29	6.6			
Uvula (C05.2)	2	0.5			
Overlapping lesion of palate or palate NOS (C05.8)	0	0.0			
Cheek mucosa (C06.0)	34	7.7			
Overlapping lesion and unspecified mouth (C06.8- C06.9)	86	19.5			
Base of tongue. NOS (C01.0–C1.09)			101	30.7	
Lingual tonsil (C02.4)			4	1.2	
Tonsil (C09)			184	55.9	
Anterior surface of epiglottis (C10.1)			3	0.9	
Lateral wall of oropharynx (C10.2)			11	3.3	
Pharynx unspecified and overlapping (C14.0)			24	7.3	
Waldeyer's ring (C14.2)			2	0.6	
Morphology	442		329	100	771
Carcinoma	41	9.3	20	6.1	61
Squamous cell	375	84.8	266	80.9	641
Adenocarcinoma	23	5.2	4	1.2	27
Lymphomas	3	0.7	39	11.9	42

With regard to morphology, the squamous forms are 84.8% for OC and 80.9% for OP: in the latter category, 11.9% of tumors are lymphomas.

The age-standardized incidence rate was 7.28 in 2020 (10.74 in males and 3.97 in females), higher than that recorded in 2019 of 5.60 (10.36 in males and 1.33 in females). Considering the sub-locations in the last period, already affected by the Covid-19 pandemic, the rates were 3.82 for OC and 3.47 for OP in 2020, respectively higher than the 2.62 and 2.97 recorded in 2019 (Table 2).

**Table 2:** Age-standardized incidence rate by subtype and sex. 2020 vs. 2019.

	2019			2020		
	*n*	Incidence rate	Age-standardized incidence rate^a^	*n*	Incidence rate	Age-standardized incidence rate^a^
Oral cavity cancer (OC)	15	2.82	2.62	22	4.15	3.82
Male	13	4.97	4.96	15	5.75	5.56
Female	2	0.74	0.56	7	2.60	2.12
Oropharyngeal cancer (OP)	17	3.20	2.97	20	3.78	3.47
Male	14	5.35	5.40	14	5.37	5.17
Female	3	1.11	0.76	6	2.23	1.85
Oral cavity and Oropharyngeal cancer (OC-OP)	32	6.02	5.60	42	7.93	7.28
Male	27	10.32	10.36	29	11.12	10.74
Female	5	1.85	1.33	13	4.83	3.97

^a^ EUROPE 2013.

**Figure 1:**
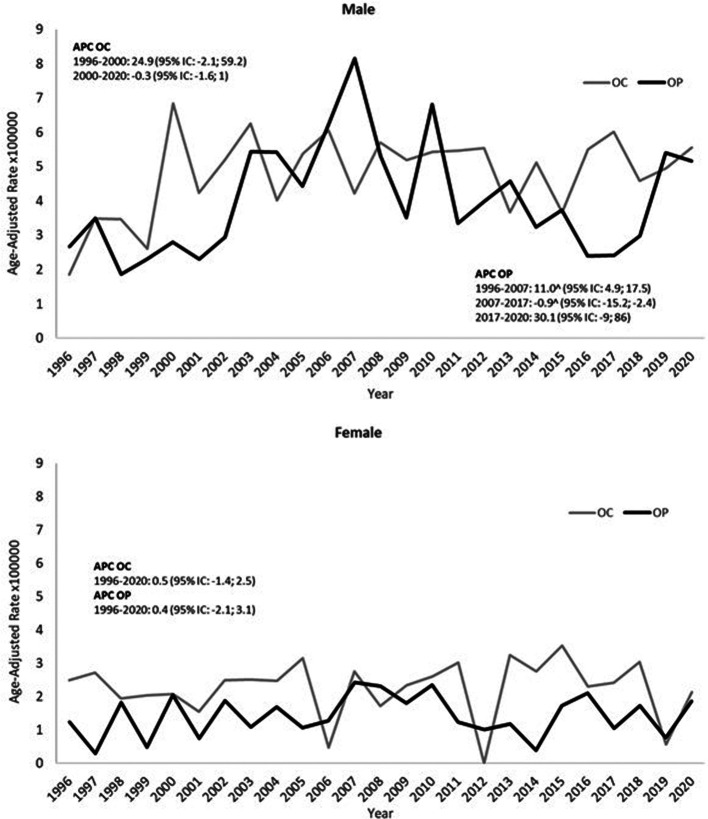
Trends in European age-standardized incidence rates of oral cavity cancer (OC) and oropharyngeal cancer (OP), males and females (1996–2020).

In males, the OP incidence trend increased statistically until 2007 and then decreased; in the last two years, we report a high number of OP cancers, but this increase is compatible with a random fluctuation. OC showed a sharp increase from 1996 to 2000 and then a plateau (Figure 1). In females, the incidence was steady over the period for both OC and OP.

**Figure 2:**
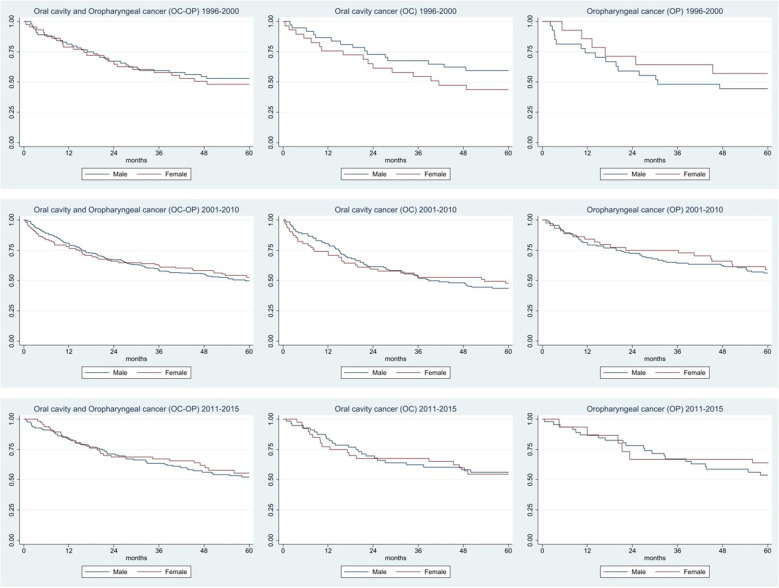
Five-year survival by subtype and sex in three different periods.

The 5-year survival for OC + OP was 51.2% (95% CI, 41.3–60.2), 50.7% (95% CI, 45.2–56.0) and 56.0% (95% CI, 47.7–63.4) in the three periods considered (Table 3 and Figure 2). For OC, survival went from 52.6% (95%CI, 39.9–63.9) to 45.1 (95% CI, 37.5–52.3) to 55.6 (95% CI, 45.0–65.0). Survival for OP goes from 48.8% (95% CI, 32.9–62.9) to 57.1% (95% CI, 48.9–64.4) to 56.5% (95% CI, 43.0–68.1).

**Table 3:** Five-year survival by subtype, sex and period. Years 1996–2015.

	1996–2000			2001–2010			2011–2015		
	5-years	IC 95%	5-years	IC 95%	5-years	IC 95%
Oral cavity cancer (OC)	52.6%	39.9%	63.9%	45.1%	37.5%	52.3%	55.6%	45.0%	65.0%
Male	59.5%	42.0%	73.2%	43.6%	34.2%	52.5%	56.3%	42.2%	68.3%
Female	43.6%	25.2%	60.6%	47.8%	34.9%	59.6%	54.7%	38.0%	68.6%
Oropharyngeal cancer (OP)	48.8%	32.9%	62.9%	57.1%	48.9%	64.4%	56.5%	43.0%	68.1%
Male	44.4%	25.6%	61.8%	56.3%	46.6%	64.9%	53.9%	38.4%	67.1%
Female	57.1%	28.4%	78.0%	59.1%	43.2%	71.9%	66.7%	37.5%	84.6%
Oral cavity and Oropharyngeal cancer (OC-OP)	51.2%	41.3%	60.2%	50.7%	45.2%	56.0%	56.0%	47.7%	63.4%
Male	53.1%	40.3%	64.4%	49.9%	43.2%	56.3%	55.1%	44.8%	64.2%
Female	48.1%	32.5%	62.1%	52.5%	42.6%	61.5%	57.8%	43.5%	69.6%

The number of prevalent live cases as of January 1, 2021 is 314 (175 OC, 139 OP): there are twice as many males as there are females. Among OC, patients with diagnoses between 2 and 9 years prevail while among OP, patients with diagnoses over 9 years prevail (Table 4).

**Table 4:** Prevalence at first January 2021 by subtype, sex and years by diagnosis.

	Years by diagnosis			All
	<2	2–9	9+	
Oral cavity cancer (OC)				
Male	23	50	39	112
Female	6	32	25	63
Oropharyngeal cancer (OP)				
Male	22	25	50	97
Female	8	16	18	42
Oral cavity and Oropharyngeal cancer (OC-OP)				
Male	45	75	89	209
Female	14	48	43	105

## Discussion

In our study, we observed a 3.8/100,000 and 3.5/100,000 incidence of oral cavity and oropharynx cancer, respectively, with an age standardized M: F ratio of approximately 2.6:1 and 2.8:1 for OC and OP, respectively. Globally, comparing the data published worldwide by GLOBOCAN (27) in 2012, the estimated age-standardized rate was about 4/100,000: 2.7 per 100,000, for OC and 1.4/100,000 for OP. The worldwide incidence is consistently higher in men than in females with a M: F ratio of 2:1 and 4.8:1 for OC and OP, respectively (28). For OP, our observed incidence is among the highest in Europe for both males and females. For OC, incidence rates are comparable with those observed in other high income countries for both males and females (28).

In males, we observed an increase in the initial period for both OC and OP, then a plateau was observed for OC, while OP significantly decreased until 2017. In females, there were no appreciable changes in incidence over the study period for both OC and OP. In the literature, the incidence of oropharyngeal cancer is rapidly increasing, especially in high-income countries and especially in the United States (29–30). In contrast, oral cavity cancer incidence rates are stable or decreasing in men globally and slightly increasing in women (31). In England, between 1995 and 2011, the OC recorded an annual increase of 2.8% in males and 3.0% in females, while for OP the APC was 7.3% in men and 6.5% in women (32). In Scotland, between 2001 and 2012, the OP increased by 85% and the OC only by 10%. These trends were found to be more pronounced in men (32–34) and in most deprived areas (33). These trends are consistent with the different relative weight of the three main risk factors in OC and OP: the prevalence of HPV infection, that is more important in OP than OC, increased in the last decades in highest income countries (35–36) and Italy (37); smoking and drinking alcohol were higher in men in the highest income countries but, at least smoking, decreased more rapidly in the last decades in men than in women (38).

Finally, it is worth noting that many studies reported an impact of the Covid-19 pandemic on the number of cancer diagnoses. In particular, a recent research carried out in Italy, in particular in the metropolitan area of Turin (39), found that compared to the seven cases expected in the period under study, only one case of oral squamous cell carcinoma (OSCC) was diagnosed. The authors suggest that this is because dentists have postponed less critical interventions (39). In our study we did not observe any decrease in the number of cancers which occurred in 2020 compared to that expected based on the incidence of previous years, as can be seen in Figure 1.

As for survival, there was a slight increase in the last period for both cancers, but this improvement was largely compatible with random fluctuation. Considering the sub-sites, survival for OC is higher in males than in females and the opposite is true for OP. In the Montero study (40), from 1960 to 2012, a survival increase of 15% was recorded (it went from 48% in the period 1960–1964 to 63% in 1985–2012). In addition, in this study it was also shown that 5-year survival ranged from 78.5% for stage I to 34.5% for stage IV (41), confirmed by rates of only 10%–40% (42–43).

It is reported in the literature that two thirds of OC (41–43) cases are diagnosed in advanced stages (stage III or IV) (43), when 5-year survival is less than 50% (44). In stages I and II, oral squamous carcinoma injuries may not cause any symptoms or discomfort; therefore, the patient does not go to the dentist or other specialists until the pathological condition becomes more serious and difficult to treat (45). Untreated patients with metastatic disease show a survival of about 4 months (41).

Another problem has been the lack of consensus in the literature on the definitions of oral cancer, often different terms have been used to identify of these neoplasms. The terminology “head and neck cancer” has also complicated the approach of the scientific and clinical community to these cancers. Oral cancer is now increasingly recognized as two different diseases: cancer of the oral cavity and of the oropharynx. This debate on definitions also emerged in a 2018 English study (4). Considering these two sites separately, OC accounts for approximately 377,000 new diagnoses and 177,000 deaths per year worldwide; OP is diagnosed about 100,000 cases a year and accounts for nearly 50,000 deaths (46–47).

As a consequence of the difficulty in the exact definition of the cases and of the terms used, the estimation of prevalent cases can also be difficult (48). Given the low incidence of the disease and its low survival rate, there are few live cases with previous diagnosis: in our province just over 300 cases. This is important, since the management of patients with oral cancer must consider not only new incident cases and pay attention to precancerous lesions, but must also consider the risk of local recurrence, which represents one of the major challenges in managing these patients.Among the limitations of the study we report that we have no indications on risk factors (smoking, alcohol and HPV infection) and we have no data on the stage of the disease.

Among the strengths, we mention the availability of 25 years of incidence, the absence of selection bias because they are population-based data and above all the availability of a year as recent as 2020, which had already been affected by the new pandemic situation.

## Conclusion

In conclusion, the availability of recent and long-term data can help clinicians and stakeholders to have greater awareness of the trend of oral cancers. Diagnostic and terminology difficulties on the one hand and changes in risk factors on the other (smoking and HPV in particular) account for the rapid changes we are observing. Covid-19, at least in our province, does not seem to have had a negative impact on new diagnoses.

## Data Availability

The raw data supporting the conclusions of this article will be made available by the authors, without undue reservation.
